# Product promotions in online supermarkets: prevalence of ‘High Fat Sugar Salt’ (HFSS) products and labelling characteristics

**DOI:** 10.1017/S1368980023001787

**Published:** 2023-11

**Authors:** Lewis W Wallis, Sally G Moore

**Affiliations:** School of Food Science and Nutrition, University of Leeds, Leeds LS3 9JT, UK

**Keywords:** Product promotions, Digital food environments, Front-of-pack nutrition labelling, High Fat Sugar Salt, Nutrient profile

## Abstract

**Objective:**

To evaluate the prevalence of ‘High Fat Sugar Salt’ (HFSS) products and front-of-pack nutrition labelling (FOPNL) characteristics across promoted products in UK online supermarkets.

**Design:**

A cross-sectional survey conducted (December 2021–January 2022) on promoted products. Data on ingredients, nutritional composition and display of FOPNL were collected from product webpages. The UK’s Nutrient Profiling Model and Multiple Traffic Light criteria were used to determine HFSS status and possession of inherent red traffic lights (iRTL), respectively. Data analysis determined the prevalence (i.e. percentage of products of the total number of products sampled) of HFSS; FOPNL and possession of iRTL. Chi-squared tests explored associations between these.

**Setting:**

Three major UK online supermarket retailer websites.

**Participants:**

Product ‘multibuy’ and ‘entrance’ promotions, from selected product categories.

**Results:**

Among the sampled 625 promoted products, the prevalence of HFSS was greater in entrance (73 %) compared with multibuy (41 %) promotions (χ^2^ (1) = 34, *P* < 0·05), with variations in the former across retailers (49–92 %). The prevalence of HFSS products in multibuy promotions offered by two retailers varied by category (i.e. Confectionery 94–97 %, Yogurts 20–20 %, Soft Drinks 16–33 %, Ready Meals 1·4–18 %). Not all promoted products displayed FOPNL on webpages (70 %) or images (52 %). A number of iRTL were found to be possessed by both HFSS and non-HFSS-promoted products.

**Conclusions:**

Prior to the 2022 implementation of Regulations restricting these, HFSS products were promoted in online supermarkets with varying display of FOPNL and possession of iRTL. Findings support future policy evaluation and mandatory digital FOPNL.

Retail food environments where food and drink products are sold either online or in-store influence consumers’ food choices such that changes to these may support improvements in health and obesity levels^(1)^. One way in which food environments can influence food choice is through various types of product promotions. These encourage consumers to purchase specific products by, for example, using a price promotion as either a discount off the original price, or by offering the product as part of a volume-based price promotion such as ‘multibuy’ deals sometimes called ‘buy-one-get-one-free’. A third type of promotion is the prominent placement of a product, including in locations at the store entrance or by the checkouts. The UK has the highest market prevalence of food products sold on promotions in Europe, with 40 % of all food and drink purchases offered in some form of promotion^(2)^.

Review evidence confirms that price promotions are likely to influence consumers’ purchasing behaviour^(3)^, leading to the purchase of more of these promoted products than originally intended^(2)^. Accordingly, the nutritional composition of products which are promoted is now a focus of new Regulations implemented from 2022^(4)^. This Regulation is part of the UK strategy to tackle rising levels of obesity and to improve intakes of nutrients of public health concern, that is, sugar, saturated fat and salt^(5)^. Specifically, the new Food (Promotion and Placement) Regulations (England) 2021 will restrict the appearance of ‘less healthy’ products within two promotional types: volume-based promotions and prominent location promotions.

## Evidence on the nutritional nature of product promotions in relation to current UK Policy

Evidence on the prevalence of price promotions on ‘healthy’ or ‘unhealthy’ products is available from a review of studies mainly focused on in-store price-reduced (discounted) products in countries outside of the UK^(3)^. This shows that in general price promotions appear to be applied more frequently to ‘less healthy’ compared with ‘more healthy’ products^(3)^. In addition, a recent meta-analysis review^(6)^ has found the global evidence is too limited and heterogeneous to be able to reach a consensus on whether price promotions are more likely to be found on healthy or unhealthy foods and drinks. A single UK-based study conducted in 2014 reported that in-store price promoted products (i.e. those with a temporary price reductions across all store branches) are more likely to be ‘less healthy’ than those which were not promoted^(7)^. Besides limited research featuring UK retail, another issue with the present evidence base is its focus on either poorly defined or price-based promotions (i.e. temporary discounts), with less emphasis on those two specific types of promotions which are to be regulated in the UK, namely ‘by volume’ (i.e. multibuy) and ‘by location’. For example, only one UK study appears to have looked at location (checkout) promotions, reporting the prevalence of less healthy products at these sites^(8)^. Another study conducted within an Irish supermarket has found that significantly more ‘multibuy’ promoted products were found to have a lower ‘nutritional quality’ score than those not promoted^(9)^. Overall, there is a lack of policy-relevant insight into the current prevalence of ‘less healthy’ products on the two types of promotions defined under current Regulations.

Further still, the current research does not fully explore UK supermarkets nor their digital food environments, providing very little insight into the prevalence of product promotions in UK supermarket websites. To our knowledge, only two studies have evaluated product and promotion data from online supermarkets, and these were based in Spain and Australia^(3)^. It is presently important to research the nutritional nature of UK online supermarket promotions given the growth of online shopping during the COVID-19 pandemic^(10)^ and the new UK Regulations which restrict promotions in this digital retail channel alongside physical stores.

## UK policy on product-level nutritional evaluation and label display

Nutritional profiling of products to classify them as either ‘healthier’ or ‘less healthy’ is a key aspect of new UK Regulations restricting promotions of the latter^(4)^. The UK Nutrient Profiling Model (NPM) is specified within these Regulations for this purpose^(11)^. This NPM uses product-level data on content of energy and selected nutrients (i.e. fibre, saturated fat, protein, Na, sugars) and some ingredients (i.e. fruit and vegetables and nuts) to compute a single overall score which determines if a product is ‘less healthy’ or in other words ‘High Fat Sugar Salt’ (HFSS). In the UK, another established way to nutritionally profile individual products is the Multiple Traffic Light (MTL) scheme, which assigns red/amber/green colours to code a product’s content of fat, saturated fat, sugars and salt as high/medium/low, respectively^(12)^. Unlike the UK NPM, the UK MTL is also a type of front-of-pack nutrition labelling (FOPNL) which can be displayed on product packaging^(12)^.

In the UK, it is a government recommendation that most pre-packaged food and drink products should voluntarily display an agreed format of FOPNL, particularly MTL^(12)^. An array of evidence^(13)^ on the impact of these labels on consumers and industry now underpins the belief that FOPNL will support improvements in public health. For example, the appearance of ‘red’ traffic lights on products may influence consumers’ food choices as well as industrial product reformulation^(14)^. In UK online supermarkets, a limited amount of previous research has revealed an inconsistent display of FOPNL, including MTL^(15)^, and also suggests that products which possess more than one inherent red traffic light (iRTL) may be less likely to display this information on their webpage^(16)^. However, there appears to be no current research into the display of FOPNL nutrition labels on promoted products (e.g. those in entrance or multibuy promotions) in UK online supermarket food environments.

This study aims to describe the prevalence of less healthy (HFSS) items as well as the FOPNL and inherent nutrition characteristics of those products within multibuy and entrance promotions on sale in UK major online supermarket retailer websites at a time point before the anticipated October 2022 introduction of the Regulations restricting these.

Specific research questions to be addressed by this study are (1) What is the current prevalence of ‘less healthy’ (HFSS) products sold in selected categories within entrance and multibuy promotions, at major UK online supermarkets? and (2) What is the prevalence of FOPNL display for these online promoted products, and how many iRTL do they possess?

## Methods

### Design and setting

A cross-sectional survey of products sold on promotion at three major UK online supermarkets was undertaken during December 2021 and January 2022. The UK’s three largest online supermarket retailers were selected based on their market share^(17)^; these were Retailer 1 – Tesco, Retailer 2 – Asda and Retailer 3 – Sainsbury’s.

**Fig. 1:**
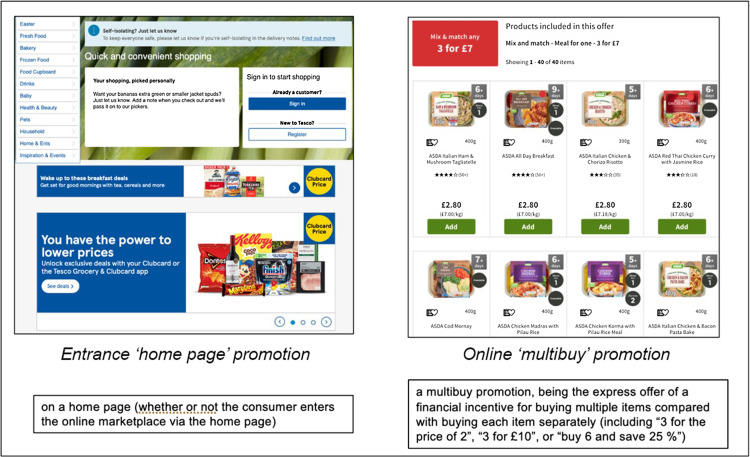
An example of one supermarket website entrance (location) (left-hand side) and their multibuy (volume) (right-hand side) promotions pages

Chosen to be specifically within the scope of the forthcoming Regulations^(4)^, both entrance and multibuy types of online product promotions were surveyed. These are defined as products promoted by (1) ‘location’ at the entrance home page (i.e. landing listings page) of the online supermarket and (2) ‘volume’ defined as those offered for a discounted price in multibuy offers (i.e. ‘3 for 2’, ‘3 for £6’) (see Fig. 1).

#### Product categories surveyed

For entrance promotions, products from all fourteen categories which are within scope of the Regulations^(18)^ were surveyed. Product types which fell outside this scope (e.g. alcohol, toiletries) were excluded. For multibuy promotions, all such products which appeared in four of the in-scope categories (i.e. Confectionery, Ready Meals (fresh and frozen), Soft Drinks and Yogurts) were included. Selection of four in-scope product categories was due to the resources available for data collection to capture all of the multibuys offered in each, and since 1000+ products were offered across all fourteen in-scope categories. The selected four categories aimed to reflect the range of major food and drink categories included in the scope of the Regulations^(4)^ which are associated with population dietary intakes of sugars, salt and saturated fats^(18)^.

### Data collection

Using a MacBook Pro (15·4-inch) laptop based in England (Yorkshire) without a personalised login, each supermarket’s online shopping website was manually accessed by the researcher (LW) in order to view promotions and access the promoted product’s individual webpages in one of two ways. For promoted products appearing on the entrance pages, individual product webpages were opened by clicking on the products images. For multibuy-promoted products, online supermarket ‘aisles’ were navigated to identify those multibuy promotions appearing in ‘special offers’ in each product category. For one retailer (Retailer 3) data were not available for multibuy promotions, which were reported to have been discontinued by this supermarket^(19)^.

**Fig. 2:**
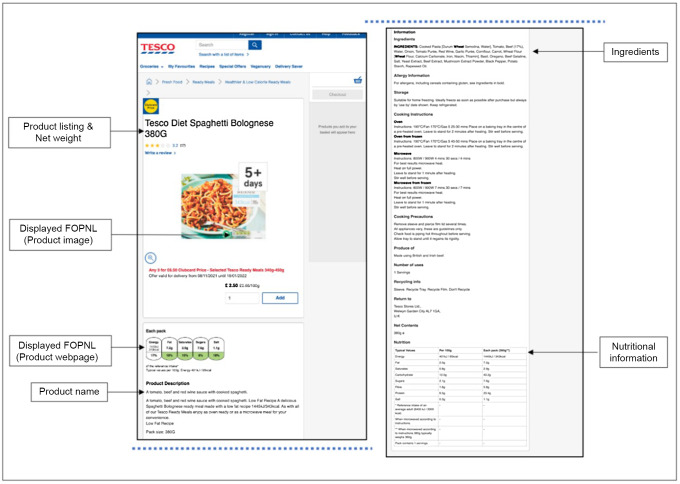
An individual product webpage from a supermarket website (split into two columns for illustration), with arrows indicating aspects of data collected. FOPNL, front-of-pack nutrition labelling

From each product webpage, data were manually collected on the following aspects: product name, price and promotional offer type, brand, ingredient declaration, serving size, and product nutrition information for ‘per 100 g’ and display and presentation format of any FOPNL (see Fig. 2). Nutrition information included content of energy (kcal/kJ), total fat (g), saturated fat (g), sugars (g) and salt (g). Where no quantitative percentage (%) information was declared for fruit, vegetable and nut ingredients, values were estimated according to other ingredient quantities, and order of appearance in the ingredient listing, using researcher judgement. Also, where any of the required nutrition information elements (e.g. fibre for which declaration is voluntary) were missing from the product webpage, values were recorded and analysed as ‘0’. For each product surveyed, the display, location and format of FOPNL on the webpage or product image (photo) were also recorded. Full product webpage screenshots were taken using the Google Chrome extension GoFullPage, for recording. Data were inputted into a Microsoft Excel (V16.52) spreadsheet for analysis.

### Data analysis

#### Application of UK Nutrient Profiling Model and Multiple Traffic Light nutrient profiling to product-level data

To evaluate each product’s UK NPM score, data analysis was conducted using Excel formulae based on the UK NPM^(11)^. Calculations used data collected on each product’s content of nutrients per 100 g (i.e. energy, protein, fibre, total sugars, saturated fat, sodium), as well as information on percentages of fruit and vegetables and nuts. In keeping with the classifications used in the UK NPM, foods were classified as ‘HFSS’ if they scored 4 or more, and drinks if they scored 1 or more^(11)^. Product NPM scores were used to calculate a mean NPM score for products within the category and by retailer which was rounded to 1 decimal place, and presented with standard deviations. Then, to evaluate the product’s content of fat, saturated fat, sugars and salt according to the MTL colour coding criteria, information on total fat, saturated fat, total sugar and salt content ‘per 100 g’ was first used with product serving size, to calculate the content of these components ‘per serving’. Then, in line with the UK MTL guidance^(13)^, delivery of each nutrient per 100 g and per serving were both used to colour code each nutrient (i.e. as green, amber or red). Following this, the number of ‘red’ traffic lights inherently possessed by each product was calculated. Those products which did not include any nutrition information on the product webpage were removed from the sample and onward analysis as nutritional evaluation could not be performed.

#### Analysis of outcomes of interest, including the prevalence of High Fat Sugar Salt

Descriptive statistics were used to describe the prevalence of HFSS products, defined as the proportion (%) of HFSS products across the sample, or according to promotion type, or retailer. The prevalence of HFSS products in multibuy promotions was also calculated within each of the four product categories. The prevalence of HFSS in entrance-promoted products was analysed across all product types since these spanned all product categories in scope of the Regulations. The prevalence of (a) products displaying FOPNL on their webpages/images and (b) products which possessed two or more iRTL was also calculated as the proportions of products with these traits. All percentages were rounded to the nearest whole number. Pearson’s chi-squared tests were used to explore associations between promotional type and proportion of HFSS *v*. non-HFSS products and the display of FOPNL on webpages or product images or proportion of products with ≥ 2 iRTL. Chi-squared statistics and *P* values are reported.

## Results

### Sample characteristics

Data were collected for 559 multibuy (182 Confectionery, 183 Ready Meals, 83 Soft Drinks, 111 Yogurts) and 106 entrance-promoted products (see Box 1). Products that did not show any nutrition information on their individual product webpages (*n* 40, i.e., 36 multibuy and 4 entrance-promoted products) were excluded from analysis, which was performed on the remaining 625 products (Box 1). Of these, 523 products were from multibuy promotions offered in the selected four product categories (Table 1). A larger proportion of the sampled multibuy-promoted products were found at Retailer 2 (*n* 343, 66 %) compared with Retailer 1 (*n* 180, 34 %). The sample also included 102 entrance promotions (see Table 1) from Retailer 1, 2 and 3, which fell across 11, 10 and 6 of the Regulation’s 14 in-scope product categories, respectively.

Box 1 Flow chart to show the collection of data from online supermarket website multibuy and entrance-promoted products. KEY: Retailer 1 – Tesco, Retailer 2 – Asda and Retailer 3 – Sainsbury’s

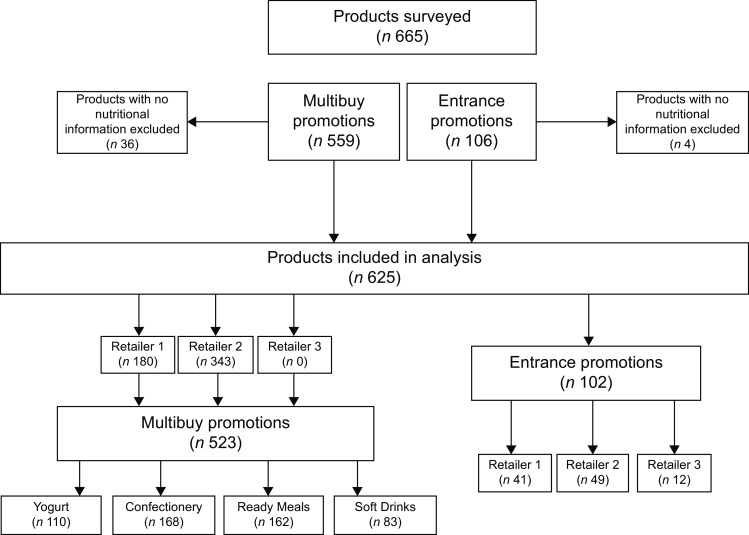

**Table 1:** Summary of sample characteristics including number of products in each promotional category, HFSS status and FOPNL characteristics

Promotional type	Number of products (percentage of products)†		Mean NPM score‡ (sd)		Number of products classified as HFSS‡ (percentage of products)§		Number of products with a specific number of inherent red traffic lights* (percentage of products)§						Number of products displaying a FOPNL on their product webpage (percentage of products)§		Number of products displaying a FOPNL on their product image (percentage of products)§	
							0		1		≥ 2					
Multibuy promotions (total all retailers)	523	(84)	6·8	(10·9)	215	(41)	220	(42)	124	(24)	179	(35)	388	(75)	277	(53)
Product category:																
Yogurt	110	(21)	0·2	(4·6)	22	(20)	100	(91)	10	(9)	0	(0)	36	(33)	8	(7)
Confectionery	168	(32)	21·0	(6·9)	160	(95)	4	(2)	51	(30)	113	(67)	112	(67)	64	(38)
Ready Meals	162	(31)	−0·03	(3·4)	18	(11)	48	(30)	48	(30)	66	(40)	160	(99)	152	(94)
Soft Drinks	83	(16)	0·2	(0·5)	15	(18)	68	(82)	15	(18)	0	(0)	80	(96)	53	(64)
Entrance promotions (total)	102	(16)	14·5	(10·6)	74	(73)	26	(26)	13	(13)	63	(62)	49	(48)	49	(48)
Retailer||:																
R1	41	(40)	8·5	(10·3)	20	(49)	20	(49)	4	(9)	17	(41)	12	(29)	27	(65)
R2	49	(48)	18·2	(9·0)	43	(88)	6	(12)	8	(16)	35	(71)	33	(67)	18	(37)
R3	12	(12)	19·5	(8·2)	11	(92)	0		1	(8)	11	(92)	4	(33)	4	(33)
Sample total	625	100 %	8·0	(11·2)	289	(46)	246	(39)	137	(22)	242	(39)	437	(70)	326	(52)

HFSS, High Fat Sugar Salt; NPM, Nutrient Profiling Model; FOPNL, front-of-pack nutrition labelling.

* Inherent red traffic lights calculated using the UK Government guidance^(13)^.

† Shown in brackets in column 2 are the percentages of the total sample or percentage of those product on either ‘multibuy’ or ‘entrance’ promotion.

‡ Products were classified as HFSS or non-HFSS according to their NPM score^(10)^, with the mean value given in column 3 rounded to 1 decimal place.

§ Shown in brackets are the percentage of products categorised according to the row heading (column 1) – i.e. by product category or retailer.

|| Retailer 1 (Tesco), Retailer 2 (Asda) and Retailer 3 (Sainsbury’s).

### Prevalence of High Fat Sugar Salt products in sampled multibuy and entrance promotions

**Fig. 3:**
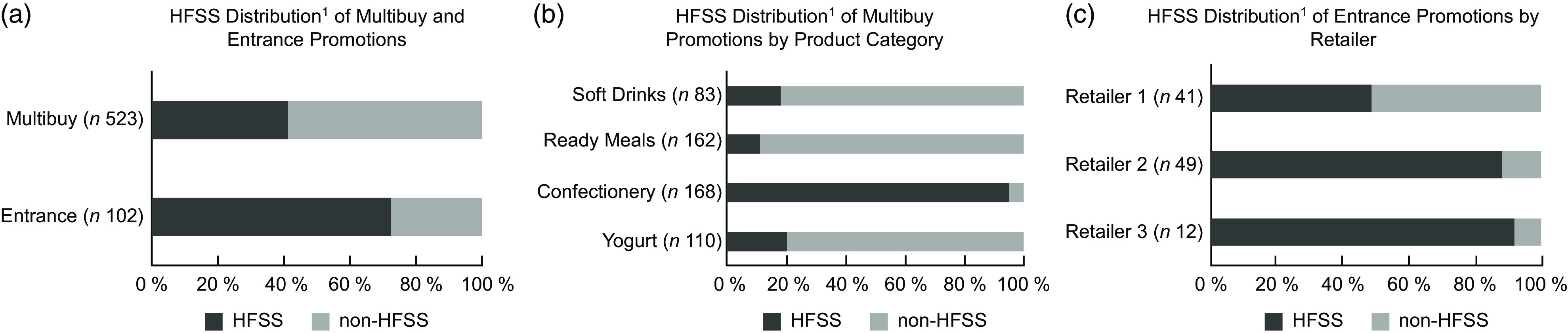
Prevalence of products classified as HFSS by (a) promotional type, (b) product category (for multibuy promotions) and (c) retailer (for entrance promotions). ^1^The UK NPM was used to calculate scores and classify ‘HFSS’ and ‘non-HFSS’ products. HFSS, High Fat Sugar Salt; NPM, Nutrient Profiling Model

The prevalence of HFSS products among sampled entrance promotions (73 %) was significantly higher than for sampled multibuy-promoted products (41 %) (χ^2^ (1) = 34·0, *P* < 0·005) (Fig. 3(a)). Within multibuy promotions, the prevalence of HFSS products was generally similar across Retailer 1 (40 %) and Retailer 2 (43 %) but varied according to product category (i.e. ranging from 95 % of Confectionery to 11 % of Ready Meals) (see Fig. 3(b)). The prevalence of HFSS in multibuy-promoted products further varied across Retailers (i.e. for Retailers 1 and 2, respectively: Confectionery 97 %, 94 %; Yogurts 20 %, 20 %; Soft Drinks 33 %, 16 %; Ready Meals 1·4 %, 18 %). Within entrance promotions, the prevalence of HFSS products ranged from 92 % (*n* 11) of those offered at Retailer 3 to 49 % (*n* 20) of those offered at Retailer 1 (Fig. 3(c)).

### Prevalence of displayed front-of-pack nutrition labelling in sampled multibuy and entrance promotions

**Fig. 4:**
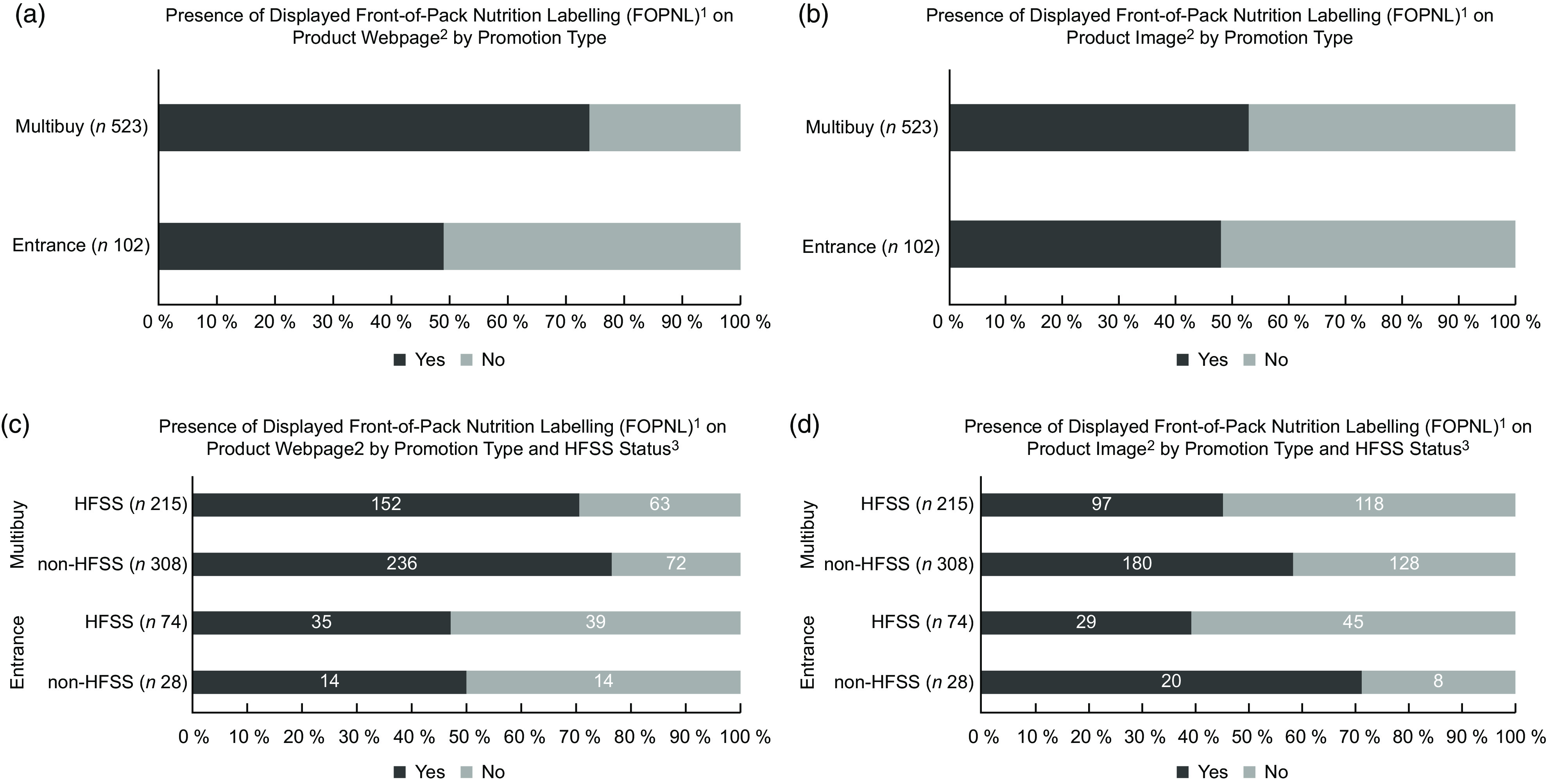
Prevalence of displayed front-of-pack nutrition labelling (FOPNL) on product webpages and product images according to promotion type (a), (b) and (c) and HFSS status (d). ^1^Displayed FOPNL included any label formats (i.e. traffic lights, monochrome labels, etc.) reflected in the Government guidance^(13)^. HFSS, High Fat Sugar Salt

FOPNL were displayed on 70 % of the sampled individual promoted product webpages and 52 % of product images (Table 1). Specifically, FOPNL appeared on 48 % of entrance and 75 % of multibuy-promoted product webpages (Fig. 4(a) and (b)) with variations according to product category and retailer (Table 1). Across all sampled promoted products, displayed FOPNL appeared on 65 % of HFSS product webpages, compared with 74 % of non-HFSS product webpages (Fig. 4(c)), although this was not statistically significant (χ^2^ (1) = 0·53, *P* = 0·47). However, compared with those products classified as non-HFSS, HFSS products were significantly less likely to display FOPNL on product images (photos) in both entrance (χ^2^ (1) = 10·4, *P* < 0·005) and multibuy promotions (χ^2^ (1) = 9·0, *P* < 0·05) (see Fig. 4(d)).

### Number of inherent red traffic lights across sampled promoted products and by High Fat Sugar Salt status

**Fig. 5:**
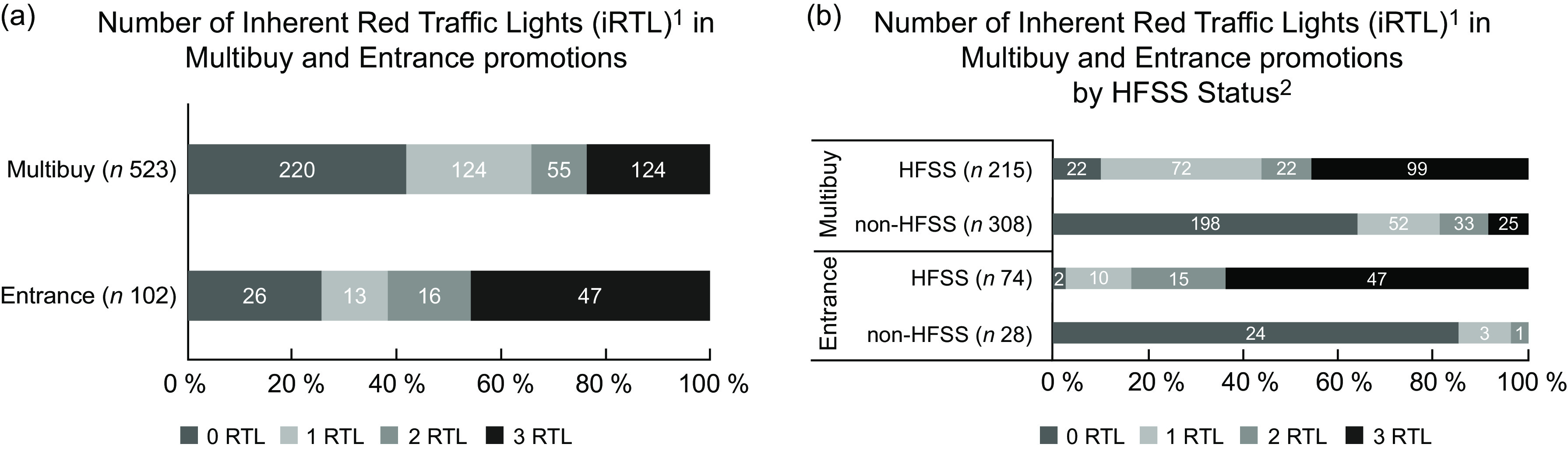
Proportions of sampled products with specific numbers of inherent red traffic lights (iRTL) (red traffic light possessed by a product, irrespective of their display) according to promotion type (a) and HFSS status of promoted products (b). HFSS, High Fat Sugar Salt

**Fig. 6:**
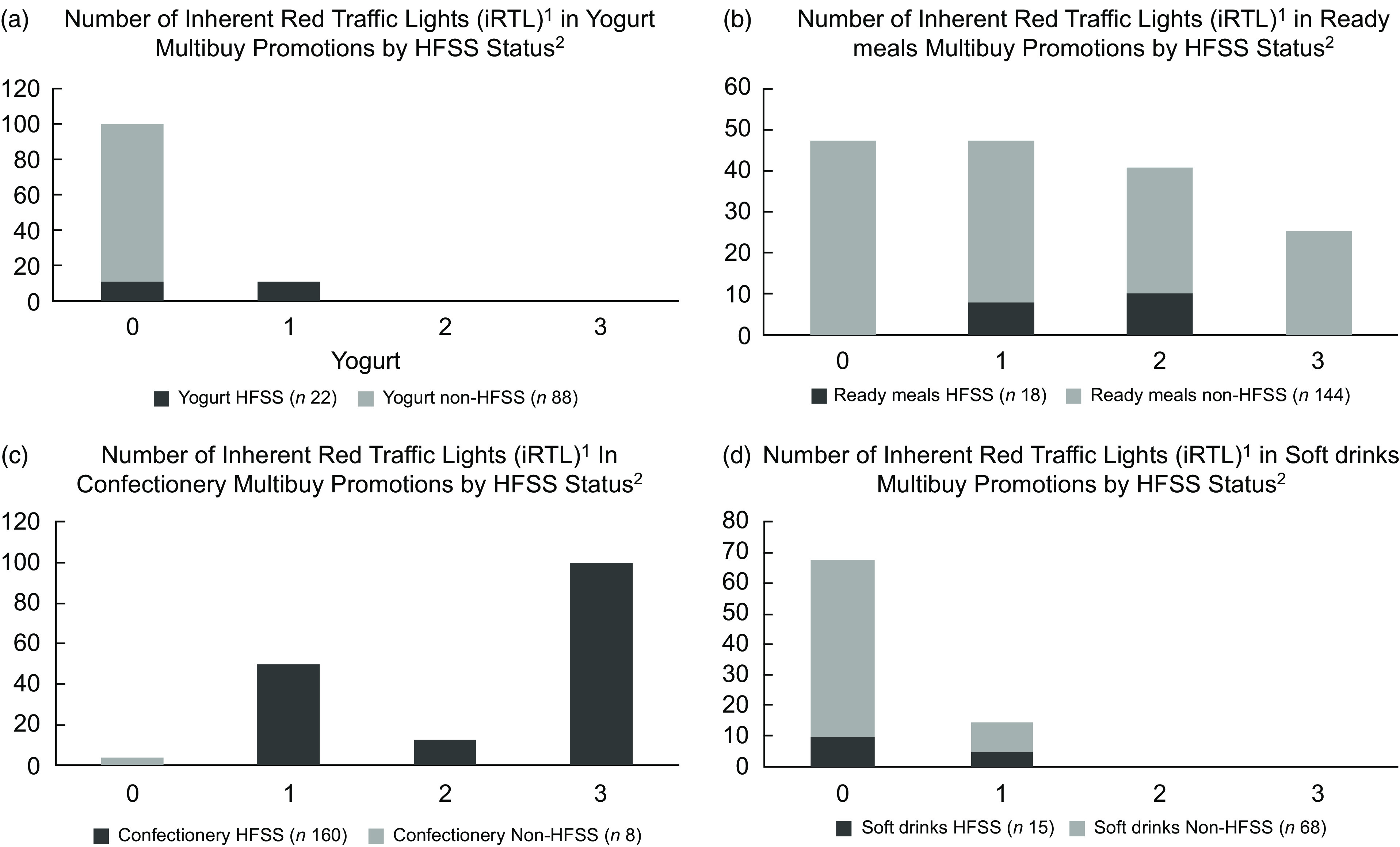
Number of inherent red traffic lights (iRTL) across products according to HFSS and non-HFSS status in multibuy promotions for (a) Yogurt, (b) Ready Meals, (c) Confectionery and (d) Soft Drinks. ^1^Red traffic lights were determined based on the Government guidance^(13)^ and ^2^each product’s HFSS status was calculated using the Nutrient Profiling Model (UK NPM)^(10)^. HFSS, High Fat Sugar Salt

Overall, 39 % of sampled promoted products possessed ≥ 2 iRTL (i.e. red traffic lights possessed by products, calculated using their declared content of fat, saturated fat, sugar and salt, irrespective of their display). This proportion varied according to FOPNL webpage/image display status, promotional type, product category and retailer (Table 1 and Figs 5 and 6). For example, FOPNL were displayed on product images/webpages on 46 %/75 % of products with ≥ 2 iRTL, and 52 %/73 % of products with less than 2 iRTL. The proportion of products with ≥ 2 iRTL was significantly greater among entrance (62 %), compared with multibuy (34 %), promotions (χ^2^ (1) = 27·2, *P* < 0·005) (Fig. 5(a)).

When examining all sampled promoted products according to their HFSS status, 63 % of HFSS products and 18 % of non-HFSS products possessed ≥ 2 iRTL. Within sampled entrance promotions, products classified as ‘less healthy’ (HFSS) had a significantly higher proportion that possessed ≥ 2 iRTL (*n* 62, 84 %) compared with sampled multibuy promotions (*n* 121, 56 %) (χ^2^ (1) = 17·9 *P* < 0·005) (Fig. 5(b)). However, there were also some non-HFSS products that possessed ≥ 2 iRTL, including 39 % of non-HFSS Ready Meals promoted via multibuy (Fig. 6).

## Discussion

### Key findings on the prevalence of High Fat Sugar Salt-promoted products, and relation to current UK policy

The first aim of this study was to evaluate the prevalence of HFSS products in sampled entrance and multibuy promotions sold in three UK supermarket websites, 8 months before the 2022 implementation of the proposed Regulations restricting these. Findings show that such ‘less healthy’ products were found in both promotional types, reinforcing the need for Regulations to restrict these. With respect to the two different promotional types, less than half (41 %) of those ‘multibuy’ (volume) promoted products (which were sampled from across four product categories: Yogurts, Ready Meals, Confectionery, Soft Drinks) were classified as HFSS, compared with over two thirds of the products sampled across entrance (location) promotions (which spanned all fourteen in-scope product categories). The prevalence of HFSS products among online entrance promotions, which also varied considerably across the three retailers in our study (i.e. ranging from 49 to 92 %), was similar to figures reported previously (35–90 %) for in-store entrance/checkout promotions in the UK^(8)^. Likewise, previous UK in-store research has reported that over 70 % of all food and drinks promoted in ‘prime’ locations (i.e. those located 10 m for store entrances, etc.) are products classified as those which ‘contribute significantly to children’s sugar and calorie intake’^(20)^. The October 2022 implementation of Regulations restricting the promotion of these specific types of products is therefore likely to considerably reduce the prevalence of these online, as well as in store, creating a level playing field for industry. The restrictions should act to reduce the current inequalities across UK supermarket online retail food environments identified here.

Another key finding is that HFSS products were found to be clearly prevalent in multibuy promotions across all four select product categories, at similar levels (around 40 %) for two of the three surveyed Retailers who offered this promotional type (the third had already reported discontinuing multibuys before the study commenced). Previous research on these types of product promotions (i.e. those multibuy deals found on flyers for a US Supermarket) has shown that a similar proportion (35 %) were classified as ‘empty calories’ using the US food-based ‘MyPlate’ classifications^(21)^. At product category level, variations in HFSS product prevalence found in our study ranged from Confectionery (95 %) to Ready Meals (11 %), with around 20 % from the Soft Drinks and Yogurt categories. Such differences are likely to reflect the composition and ingredients of products in each category given the application of the UK NPM is not category specific and uses a standard 100 g reference amount. Additionally, the proportion of HFSS products in each category can affected by reformulation undertaken by manufacturers, such as those seen following UK policy initiatives to reduce sugar^(22)^, which is one parameter of the UK NPM score. In comparison, data from a two major retailers in Australia (from products available online in 2016) show that over half (52–59 %) of multibuy-promoted products were from the sugar-sweetened beverage category (i.e. Soft Drinks, fruit-flavoured drinks, etc.)^(23)^.

While now delayed by the UK Government^(24)^, restrictions to HFSS multibuy promotions should in future act to eliminate these type of products across all three retailers and specific product categories. Our findings also imply that the delay in the implementation of these restrictions will mean that inequalities across retailers in their multibuy HFSS product promotions will likely continue across major UK online supermarket retail food environments^(25)^. This is important given the range of shopper socio-demographic backgrounds which are likely served by each of the online retailers surveyed here. Limited insight on this indicates at least one of the included retailers is known to be used by more affluent consumers^(26)^. However, it should be highlighted that emerging evidence also indicates there may be inequalities in online supermarket access and delivery availability, since these may be lower for consumers living in more deprived areas^(27)^.

### Key findings on front-of-pack nutrition labelling and inherent red traffic light characteristics of promoted products, including by High Fat Sugar Salt status, and relation to current UK Policy

Second, this research aimed to describe the prevalence of displayed FOPNL across these promoted products’ webpages and images (photos). Findings show that display of FOPNL is not fully provided across the sampled promoted products’ webpages and images (photos), including for both HFSS and non-HFSS products, and may be favoured on non-HFSS product images. While variable prevalence is perhaps an inevitable consequence of the voluntary nature of the UK FOPNL Government recommendation^(12)^, our findings are also supported by other indications of a reluctance from retailers to label ‘less healthy’ products with a FOPNL on product packaging^(16)^. Despite the need for this information to help consumers make healthier choices, our findings reflect previous findings showing incomplete market penetration of FOPNL display on product webpages in UK^(28)^ and international^(29)^ online supermarket settings. Our work is believed to be the first investigation of FOPNL display among online product promotions and also in relation to product HFSS status.

Finally, we sought to evaluate HFSS/non-HFSS-promoted products in relation to the number of iRTL they possess, according the UK MTL criteria which guides those colours displayed on this format of FOPNL. To our surprise, findings indicate that both non-HFSS and HFSS-promoted products possessed ≥ 2 iRTL, including around a third of non-HFSS multibuy-promoted Ready Meals. This work therefore highlights a concern that promoted products which are non-restricted (i.e. non-HFSS) may also possess a number of iRTL, which may or not be displayed. Indeed, a product’s HFSS status is not displayed, nor easy for shoppers to check (i.e. using the UK NPM), which therefore necessitates the use of FOPNL, including MTL, as a means to evaluate product-level healthfulness.

### Strengths and limitations

Limitations exist in the methodology used here, the first of which concerns the representativeness of the findings given the limited number of online retailers and products sampled in the retail period of time from December to January. This sampling approach reflected the initial exploratory nature and resourcing of this project, and the large market share of each the retailers included. It should also be noted that data collection was conducted during both the COVID-19 pandemic and a seasonal time period, during which UK retailers are known to present shoppers with various promotional campaigns and product innovations (i.e. Christmas, healthier eating in January, etc.). While different ranges of products are sold during these periods, it should still be noted that any entrance/multibuy promotions associated with the product categories included here are nevertheless considered in scope of the new Regulations.

Another strength of the study is that all of the included product categories and specific entrance/multibuy promotional types were in scope of the new Regulations. These were ascertained following careful scrutiny of the Government Consultation^(17)^ and available guidance on the Regulations’ implementation^(4)^. The approach used here therefore enables future policy impact evaluation. However, limitations on time and resource meant that we sampled all multibuy-promoted products in four of the fourteen in-scope product categories. This inevitably meant that some other types of products which are also in-scope of the future restrictions on multibuy product promotion were not sampled here, for example, those with a likely high prevalence of HFSS products such as ‘Cakes and Cupcakes (Category 6)’. This approach has therefore given an initial snapshot of the overall prevalence estimates for HFSS product promotions for the sampled multibuy promotions, but further work on wider selection of product types is warranted.

Our product sample size was limited by our manual approach to data collection, as opposed to automatic scraping. Manually collecting data did however enable the collection of data on several specific consumer-relevant aspects, including locating the display of FOPNL on product webpages and images, which may not have been possible using automated processes in the time frame available. Another limitation was the quality of the available product-level data collected from individual product webpages which was required in order to calculate each product’s NPM score and classify their HFSS status. As reported, some necessary ingredient and nutrition label information was not available on some product’s webpages. This meant that some ingredient amounts were estimated by researchers, some nutrition elements (i.e. fibre) were analysed using ‘0’ or where no nutrition information was declared products were removed from the sample. For the former two, these were thought to have a limited impact on our findings given the anticipated content of specific fruit/vegetable ingredients or fibre content of the (i.e. Soft Drinks) products is likely to be low and hence unlikely to be scored within the UK NPM thresholds (which are 40 %) for these components. Overall, such online supermarket product-level data limitations mean there currently exists the potential for products to be misclassified using the UK NPM. This is an issue which has been previously raised by researchers in the area of in-store product information^(30)^ and by CODEX in relation to information provision in e-commerce^(31)^.

### What the work adds to the evidence and implications for policy

Overall, to our knowledge, our study is the first to show that promotions (i.e. by volume and location) in the online supermarkets include those ‘less healthy’ products currently targeted by the Regulations due to be implemented after this study was conducted. This work adds to the existing international literature reporting the prevalence of promotions of ‘less healthy’ products^(3)^ by examining the prevalence of HFSS products across two specifically defined promotional types (i.e. entrance and multibuy) in UK online supermarkets. Our use of the UK NPM^(11)^ specifically reflects that this tool is used in the new Regulations restricting promotions to classify ‘less healthy’ (HFSS) foods and drink products. It also responds to the acknowledged challenges faced by researchers reviewing those previous studies across which there is a lack of continuity on the types of nutritional evaluation and profiling tools researchers have used to evaluate product-level healthfulness^(32)^. Indeed, a recent NHS Scotland report has identified a lack of UK studies reporting the nutritional profile of multibuy-promoted products^(33)^, while other research has heterogeneously categorised ‘healthy’ and ‘unhealthy’ products via their type, or with reference to general food-based dietary guidance (i.e. ‘core’ *v*. ‘discretionary’ foods)^(3,6,34)^. Implications for policy makers working to enable healthier retailer food environments^(35)^ begin with fully appreciating the need for a product-level nutrient profiling approach to policy evaluation and enforcement, described herein. Specifically, it is key to note that, under the Regulations, products are expected to be internally classified as ‘HFSS’ (i.e. by manufacturers) in a process which requires detailed data on the product’s recipe and nutritional content for use within the UK NPM.

Our findings also show that there is clearly a need for continued evaluation of HFSS prevalence in specific product and promotional types in online supermarkets. Such evaluation should now also seek to encompass the impact of the delayed restrictions on HFSS multibuy product promotions and any other changes to promote healthier food choice in online supermarket websites. Ultimately, findings suggest that, for industry, replacing promoted less healthy (HFSS) products in the retail promotion space with healthier types such as fruit and vegetables could be an effective means of supporting healthier food choices, as indicated by a recent (in-store) retail trial undertaken with the industry^(36)^. Implementation of the Regulations extends also beyond retailers themselves to manufacturers and brands, who can determine the nature of product promotions in online supermarkets.

This work also provides the first insight on the display of FOPNL specifically across promoted non-HFSS/HFSS products, lacking in the research literature, including for the online shopping retail channel^(1)^. From both a consumer and policy perspective, our findings support a need to mandate FOPNL for reliable presentation to consumers in UK online shopping/digital food environments where they have potential to influence consumer choices^(37)^, including across non-HFSS products which may still possess a number of iRTL. Policy makers should note opportunities to mandate display of FOPNL online, including the (2020) UK Government consultation on the future of FOPNL post-BREXIT^(38)^. Finally, policy makers, industry and consumers should be aware that our findings also highlight a potential discrepancy between the UK NPM and MTL, two policy-relevant profiling tools which are currently used in the UK to evaluate product-level ‘healthfulness’. Since each uses a non-category specific, slightly different nutrient/energy and ‘per 100 g’ or/and ‘per serving’ parameters, there is potential for products to be both non-HFSS while possessing inherent ‘red’ traffic lights.

## Conclusions

Findings from this work are thought to be the first to show the varied prevalence of ‘less healthy’ HFSS products promoted in ‘multibuy’ and ‘entrance’ promotions across UK online supermarket retailers and product categories, before the implementation of Regulations restricting these. Furthermore, the incomplete display of FOPNL across promoted HFSS and non-HFSS products means that consumers are not reliably provided with this information to help steer their healthier food choices. This is of further concern given that the UK NPM, whose scores determine the HFSS status of a product, differs slightly from the UK MTL colour coding criteria leading to some ‘non-HFSS’ may also be inherently red (high) in specific nutrients of public health concern. Findings now offer a baseline for future work to evaluate the impact of the UK Regulations restricting such products from promotions from October 2022 onwards, and evidence to support mandating FOPNL within these digital food environments.

## References

[1] Wyse R , Jackson JK , Delaney T et al. (2021) The effectiveness of interventions delivered using digital food environments to encourage healthy food choices: a systematic review and meta-analysis. Nutrients 13, 2255–2263. 34208869 10.3390/nu13072255 PMC8308236

[2] Public Health England (2015) Sugar Reduction: The Evidence for Action Annex 4: An Analysis of the Role of Price Promotions on the Household Purchases of Food and Drinks High in Sugar. https://assets.publishing.service.gov.uk/government/uploads/system/uploads/attachment_data/file/470175/Annexe_4._Analysis_of_price_promotions.pdf (accessed November 2022).

[3] Bennet R , Zorbas C , Huse O et al. (2022) Prevalence of healthy and unhealthy food and beverage price promotions and their potential influence on shopper purchasing behaviour: a systematic review of the literature. Obes Rev 21, e12948. 10.1111/obr.12948 31633289

[4] Department of Health and Social Care (2022) Restricting Promotions of Products High in Fat, Sugar or Salt by Location and by Volume Price: Implementation Guidance. https://www.gov.uk/government/publications/restricting-promotions-of-products-high-in-fat-sugar-or-salt-by-location-and-by-volume-price/restricting-promotions-of-products-high-in-fat-sugar-or-salt-by-location-and-by-volume-price-implementationguidance (accessed December 2022).

[5] Department of Health and Social Care (2022) Tackling Obesity: Empowering Adults and Children to Live Healthier Lives. UK Government. https://www.gov.uk/government/publications/tackling-obesity-government-strategy/tackling-obesity-empowering-adults-and-children-to-live-healthier-lives (accessed September 2022).

[6] Kaur A , Lewis T , Lipkova V et al. (2020) A systematic review, and meta-analysis, examining the prevalence of price promotions on foods and whether they are more likely to be found on less-healthy foods. Public Health Nutr 23, 1281–1296. 32209142 10.1017/S1368980019004129 PMC7196736

[7] Nakamura R , Suhrcke M , Jebb SA et al. (2015) Price promotions on healthier compared with less healthy foods: a hierarchical regression analysis of the impact on sales and social patterning of responses to promotions in Great Britain. Am J Clin Nutr 101, 808–816. 25833978 10.3945/ajcn.114.094227 PMC4381774

[8] Ejlerskov K , Sharp S , Stead M et al. (2018) Supermarket policies on less-healthy food at checkouts: natural experimental evaluation using interrupted time series analyses of purchases. PLoS Med 15, e1002712. 30562349 10.1371/journal.pmed.1002712 PMC6298641

[9] SafeFood (2019) What’s on Offer? The Types of Food and Drink on Price Promotion in Retail Outlets in the Republic of Ireland. www.safefood.net (accessed December 2022).

[10] East R (2021) Online grocery sales after the pandemic. Int J Mark Res 64, 13–18.

[11] Department of Health and Social Care (2011) The Nutrient Profiling Model https://www.gov.uk/government/publications/the-nutrient-profiling-model (accessed May 2022).

[12] Department of Health (2016) Guide to Creating a Front of Pack (FoP) Nutrition Label for Pre-Packed Products Sold through retail outlets. https://www.food.gov.uk/sites/default/files/media/document/fop-guidance_0.pdf (accessed July 2022).

[13] Nohlen H , Bakogianni I , Grammatikaki E et al. (2022) Front-of-pack Nutrition Labelling Schemes: An Update of the Evidence, EUR 31153 EN. Luxembourg: Publications Office of the European Union. ISBN 978–92–76–55032–7. doi: 10.2760/932354,JRC130125. https://publications.jrc.ec.europa.eu/repository/handle/JRC130125) (accessed September 2022).

[14] Scarborough P , Matthews A , Eyles H et al. (2015) Reds are more important than greens: how UK supermarket shoppers use the different information on a traffic light nutrition label in a choice experiment. Int J Behav Nutr Phys Act 12, 1–9. 26652916 10.1186/s12966-015-0319-9 PMC4676872

[15] Stones C (2016) Online food nutrition labelling in the UK: how consistent are supermarkets in their presentation of nutrition labels online? Public Health Nutr 12, 2175–2184. 10.1017/S1368980015003110 PMC10270821 26553334

[16] Hall A & Moore SG (2021) Penetration and presentation of front-of-pack nutrition labelling in UK supermarket websites: preliminary survey results. J Epidemiol Community Health 75, P24.

[17] Department for Health and Social Care (2020) Impact Assessment: Restricting Volume Promotions for High Fat, Sugar, and Salt (HFSS) Products. https://assets.publishing.service.gov.uk/government/uploads/system/uploads/attachment_data/file/1008423/impact-assessment-restricting-checkout-end-of-aisle-and-store-entrance-sales-of-HFSS.pdf) (accessed November 2022).

[18] Department of Health and Social Care (2021) Restricting Promotion of Products High in Fat, Sugar and Salt by Location and by Price: Government Response to Public Consultation. https://www.gov.uk/government/consultations/restricting-promotions-of-food-and-drink-that-is-high-in-fat-sugar-and-salt/outcome/restricting-promotions-of-products-high-in-fat-sugar-and-salt-by-location-and-by-price-government-response-to-public-consultation (accessed November 2021).

[19] Sainsbury’s (2016) Sainsbury’s to Phase Out Multi-Buy Promotions in Favour of Lower Regular Prices. www.about.sainsburys.co.uk (accessed December 2022).

[20] Obesity Health Alliance (2017) Out of Place Report. The Extent of Unhealthy Foods in Prime Locations in Supermarkets. www.obesityhealthalliance.org.uk (accessed November 2022).

[21] Exum B , Thompson SH & Thompson L (2014) A pilot study of grocery store sales: do low prices = high nutritional quality? NutrFood Sci 44, 64–70.

[22] Scarborough P , Adhikari V , Harrington RA et al. (2020) Impact of the announcement and implementation of the UK Soft Drinks Industry Levy on sugar content, price, product size and number of available soft drinks in the UK, 2015–2019: a controlled interrupted time series analysis. PLoS Med 17, e1003025. 32045418 10.1371/journal.pmed.1003025 PMC7012398

[23] Zorbas C , Gilham B , Boelsen-Robinson T et al. (2019) The frequency and magnitude of price-promoted beverages available for sale in Australian supermarkets. Aust N Z J Public Health 43, 346–351. 31180614 10.1111/1753-6405.12899

[24] Department of Health and Social Care (2022) Government Delays Restrictions on Multibuy Deals and Advertising on TV and Online. Press Release 14th May 2022. https://www.gov.uk/government/news/government-delays-restrictions-on-multibuy-deals-and-advertising-on-tv-and-online) (accessed May 2022).

[25] Moore SG & Butler T (2022) UK government delays restriction of promotions on less-healthy foods: serious implications for tackling obesity. Obesity 30, 1722–1723. 35899320 10.1002/oby.23524 PMC9546114

[26] Clark SD , Shute B , Jenneson V et al. (2021) Dietary patterns derived from UK supermarket transaction data with nutrient and socioeconomic profiles. Nutrients 13, 1481. 33925712 10.3390/nu13051481 PMC8147024

[27] Urquhart R , Newing A , Hood N et al. (2022) Last-mile capacity constraints in online grocery fulfilment in Great Britain. J Theor Appl Electron Commer Res 17, 636–651.

[28] Ogundijo DA , Tas AA & Onarinde BA (2021) An assessment of nutrition information on front of pack labels and healthiness of foods in the United Kingdom retail market. BMC Public Health 21, 220–228. 33550987 10.1186/s12889-021-10255-4 PMC7868120

[29] Olzenak K , French S , Sherwood N et al. (2020) How online grocery stores support consumer nutrition information needs. J Nutr Educ Behav 52, 952–957. 33039023 10.1016/j.jneb.2020.07.009 PMC7538868

[30] Jenneson V & Morris MA (2021) Data considerations for the success of policy to restrict in-store food promotions: a commentary from a food industry nutritionist consultation. Nutr Bull 46, 40–51.

[31] FAO/WHO Food Standards Programme Codex Committee on Food Labelling (2021) Draft Guidelines on Internet Sales/E-Commerce. https://www.fao.org/fao-who-codexalimentarius/sh-proxy/en/?lnk=1&url=https%253A%252F%252Fworkspace.fao.org%252Fsites%252Fcodex%252FMeetings%252FCX-714-46%252Ffl46_07e.pdf (accessed March 2023).

[32] Maganja D , Miller M , Trieu K et al. (2022) Evidence gaps in assessments of the healthiness of online supermarkets highlight the need for new monitoring tools: a systematic review. Curr Atheroscler Rep 4, 215–233. 10.1007/s11883-022-01004-y PMC9023389 35138570

[33] Martin L , Bauld L & Angus K (2017) Rapid Evidence Review: The Impact of Promotions on High Fat, Sugar and Salt (HFSS) Food and Drink on Consumer Purchasing and Consumption Behaviour and the Effectiveness of Retail Environment Interventions. Edinburgh: NHS Health Scotland. https://www.healthscotland.scot/media/1611/rapid-evidence-review-restriction-of-price-promotions.pdf

[34] Riesenberg D , Backholer K , Zorbas C et al. (2019) Price promotions by food category and product healthiness in an Australian supermarket chain, 2017–2018. Am J Public Health 109, 33–45. 10.2105/AJPH.2019.305229 PMC6727276 31415196

[35] Fernandez MA & Raine KD (2021) Digital food retail: public health opportunities. Nutrients 13, 3789. 34836044 10.3390/nu13113789 PMC8624168

[36] IGD (2021) Healthy, Sustainable Diets: Driving Change. https://www.igd.com/articles/article-viewer/t/driving-change/i/29113 (accessed January 2023).

[37] Jusic N , Fagerstrøm A , Pawar S et al. (2022) Effects of digitalized front-of-package food labels on healthy food-related behavior: a systematic review. Behav Sci 12, 363. 36285932 10.3390/bs12100363 PMC9598805

[38] Department of Health and Social Care, Department of Health (Northern Ireland) & The Scottish Government, and Welsh Government (2020) Consultation: Front-of-Pack Nutrition Labelling in the UK: Building on Success. ’https://www.gov.uk/government/consultations/front-of-pack-nutrition-labelling-in-the-uk-building-on-success#:∼:text=Consultation%20description&text=The%20Welsh%20version%20of%20the,way%20that’s%20easy%20to%20understand) (accessed December 2022).

